# Orbital myiasis in a case of invasive basal cell carcinoma

**DOI:** 10.4103/0974-620X.48422

**Published:** 2009

**Authors:** U. K. Raina, M. Gupta, V. Kumar, B. Ghosh, R. Sood, S. A. Bodh

**Affiliations:** Guru Nanak Eye Centre, Maulana Azad Medical College, Maharaja Ranjeet Singh Marg, New Delhi, India

Myiasis is the invasion of living animal tissue by fly larvae (maggots). Ocular myiasis or ophthalmomyiasis is the term used when larvae invade the eye. It is known as external ophthalmomyiasis when larvae attack the lids or conjunctiva and internal ophthalmomyiasis when the globe is invaded. Ocular myiasis in humans was first reported by Keyt in 1900[1] the first case from India was described by Elliot in 1910.[1]

Ophthalmomyiasis is usually caused by larvae of the sheep nose botfly (Oestrus ovis),[2] and less commonly by human botfly (Dermatobia hominis). The flies themselves do not parasitize the host. The adult flies give birth to live young (larvae) which are capable of parasitizing hosts immediately. The larvae are capable of living in eye fluid, freely crawling on the eyeballs with the help of hooks and tend to attach themselves to the conjunctiva and erode the mucous membrane of the eye.

Ophthalmomyiasis is seen to occur in eyes of humans, living or working in close proximity to livestock. Children, older people, immuno-compromised patients with orbital carcinomas, diabetics, and patients on immunosuppressive therapy are usually affected. However, few reports exist, which show the infestation even among non compromised hosts.[3] Poor environmental sanitation and personal hygiene are often considered as responsible factors. Infestation occurs by contaminated fingers of the patient after handling the infested cattle or the fly itself. Beneath the skin, the larvae start eating the tissue and grow into large maggots.

**Figure 1:**
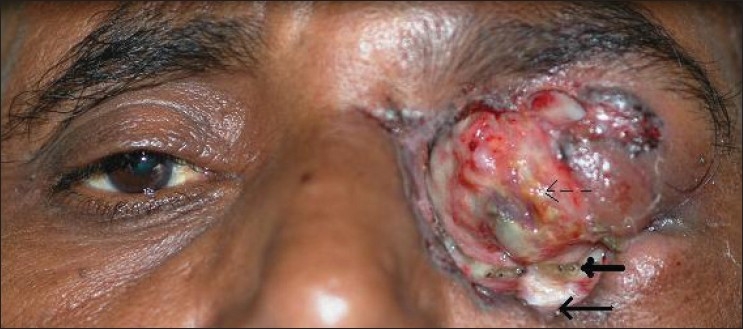
Orbital myiasis thick solid arrow: Pigmented margin of the lesion; which was the biopsy site. Thin solid arrow: Larvae, yellow-brown in color. Broken arrow: the disorganized globe

**Figure 2:**
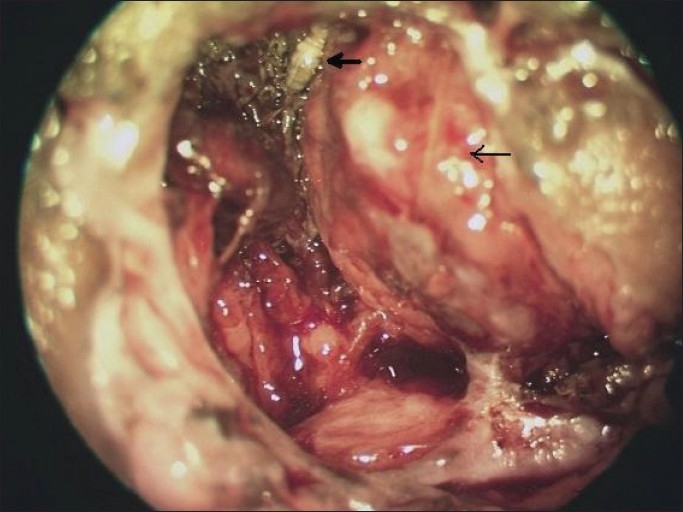
Maggots infesting the orbit (Higher Magnification) Thick arrow: larvae seen as white shiny structures. Thin arrow: showing disorganized globe

The patient, a 50-year-old man, and a wild stock farmer by occupation, presented with a brown, fleshy, ulcerated, and foul-smelling lesion involving the left orbit. The patient gave a history of a gradually increasing mass from the lower lid, of six months duration. No previous treatment had been sought. Examination under magnification revealed the mass to be the destroyed globe. No eyelids were identified. The orbital margin showed pigmentation of the skin. Multiple maggots [Figures 1 and 2] were seen to be infesting the lesion with excessive mucopurulent discharge.

**Figure 3:**
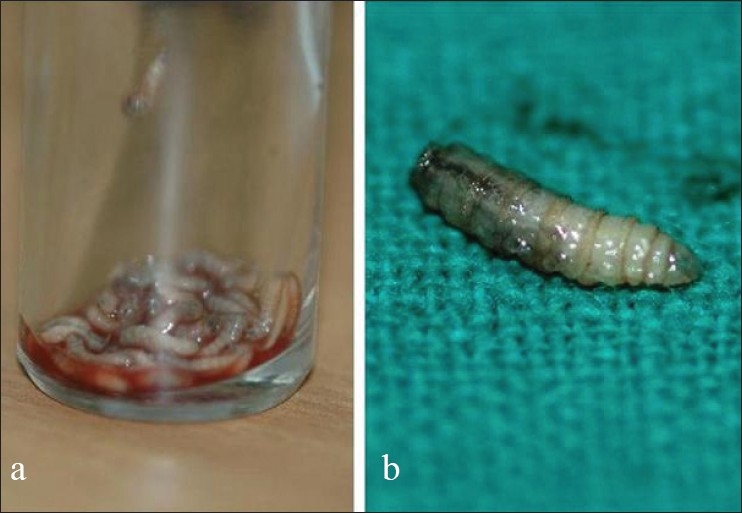
Larvae. a. Larvae extracted from the lesion, transported in a clean sterile container for entomological assessment. b. Magnified view of the chitinous body structure of larvae

The larvae were manually removed from the superficial tissues [Figure 3] with a forceps after immobilizing them by applying 4% xylocaine. Following this, turpentine oil painting was done with which the maggots came out of the deeper tissues and could be removed easily. The collected larvae [Figure 3] were sent to entomology for micro slide preparation. The maggots were identified as belonging to Phylum Arthropoda, Class Insecta, Subclass Pterygota, Order Diptera, Subsection Calyptratae, and Family Calliphoridae. Bacteriological study of conjunctival secretion did not reveal any abnormality. Sterile dressings were applied and systemic antibiotics were given along with analgesics.

Histopathology from margins of the ulcerated lesion revealed Basal Cell Carcinoma. Computed tomography (CT) imaging revealed complete destruction of the left orbit with invasion of the ipsilateral maxillary sinus but no evidence of any intracranial spread. A thorough systemic workup revealed no metastasis. Biochemical evaluation including blood sugars, cell counts, hemogram with platelet counts, and urine analysis was normal.

**Figure 4:**
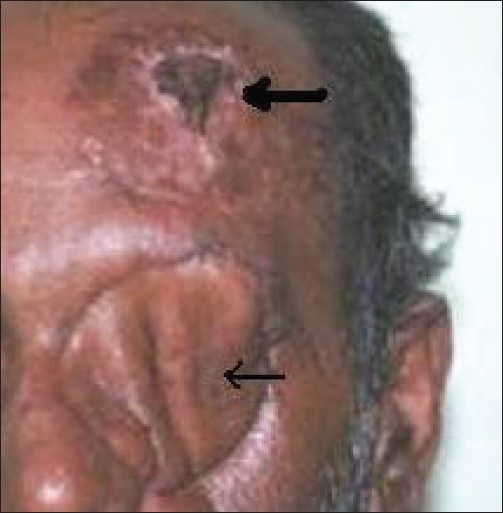
Final result after exenteration of the orbit with forehead flap reconstruction (thick arrow: donor site, thin arrow: rotational flap)

The patient underwent a section IV exenteration procedure in conjunction with otolaryngologist with reconstruction using forehead skin flap. The patient was followed up for 6 months with no further complaints. The donor site for the forehead flap healed well with good take of the flap [Figure 4].

Ophthalmomyiasis is an uncommon clinical condition, with isolated case reports in world literature[4] and human infestation with Family Calliphoridae is rare.
